# Heat stress causes dysfunctional autophagy in oxidative skeletal muscle

**DOI:** 10.14814/phy2.13317

**Published:** 2017-06-23

**Authors:** Alexandra J. Brownstein, Shanthi Ganesan, Corey M. Summers, Sarah Pearce, Benjamin J. Hale, Jason W. Ross, Nicholas Gabler, Jacob T. Seibert, Robert P. Rhoads, Lance H. Baumgard, Joshua T. Selsby

**Affiliations:** ^1^ Department of Animal ScienceIowa State UniversityAmesIowa; ^2^ Department of Animal and Poultry SciencesVirginia TechBlacksburgVirginia

**Keywords:** Heat stroke, hyperthermia, mitochondria, mitophagy, oxidative stress

## Abstract

We have previously established that 24 h of environmental hyperthermia causes oxidative stress and have implicated mitochondria as likely contributors to this process. Given this, we hypothesized that heat stress would lead to increased autophagy/mitophagy and a reduction in mitochondrial content. To address this hypothesis pigs were housed in thermoneutral (TN; 20°C) or heat stress (35°C) conditions for 1‐ (HS1) or 3‐ (HS3) days and the red and white portions of the semitendinosus collected. We did not detect differences in glycolytic muscle. Counter to our hypothesis, upstream activation of autophagy was largely similar between groups as were markers of autophagosome nucleation and elongation. LC3A/B‐I increased 1.6‐fold in HS1 and HS3 compared to TN (*P* < 0.05), LC3A/B‐II was increased 4.1‐fold in HS1 and 4.8‐fold in HS3 relative to TN, (*P* < 0.05) and the LC3A/B‐II/I ratio was increased 3‐fold in HS1 and HS3 compared to TN suggesting an accumulation of autophagosomes. p62 was dramatically increased in HS1 and HS3 compared to TN. Heat stress decreased mitophagy markers PINK1 7.0‐fold in HS1 (*P* < 0.05) and numerically by 2.4‐fold in HS3 compared to TN and BNIP3L/NIX by 2.5‐fold (*P* < 0.05) in HS1 and HS3. Markers of mitochondrial content were largely increased without activation of PGC‐1*α* signaling. In total, these data suggest heat‐stress‐mediated suppression of activation of autophagy and autophagosomal degradation, which may enable the persistence of damaged mitochondria in muscle cells and promote a dysfunctional intracellular environment.

## Introduction

Every homeothermic organism has a set temperature range, known as a thermoneutral zone, in which they can adequately maintain an equilibrium of endogenous and exogenous heat production and loss (Swanlund et al. 2008). Heat stress occurs when there is a net gain in thermal energy beyond the thermoneutral zone (Fuquay 1981; Swanlund et al. 2008; DeShazer et al. 2009). Given recent and predicted environmental changes there is a likelihood of recurrent, intense heat events that will ostensibly lead to an increased frequency and severity of heat stress (Hajat et al. 2014; Macpherson 2014; Gamble et al. 2008). Importantly, heat stress poses a pressing and urgent threat to human and animal health and wellbeing as well as agricultural economics and food security (Fuquay 1981; MKilbourne 1997; St.‐Pierre et al. 2003; Haines et al. 2006; DeShazer et al. 2009; Macpherson 2014). Despite these significant health, humanitarian, and economic concerns, there are no available remedies to treat heat‐mediated pathologies aside from hydration and cooling (Aberle et al. 1974; Fuquay 1981; St.‐Pierre et al. 2003; DeShazer et al. 2009). Moreover, the cellular mechanisms by which heat‐stress‐related injuries occur are largely unknown. Improving our understanding of systemic and cellular pathophysiology is necessary in order to develop appropriate prophylactics and interventions.

Pathologic changes caused by heat stress are in stark contrast to events resulting from therapeutic hyperthermia where blunted atrophy (Selsby and Dodd 2005), enhanced regrowth and hypertrophy (Selsby et al. 2007; Baumgard and Rhoads 2013), and increased insulin sensitivity (Gupte et al. 2011) have been observed following brief heat exposures. Further, heat stress also appears to be distinct from physiological changes occurring during heat stroke, which generally includes a hyperthermic exposure of less than 2 h often compounded by exercise (Leon and Bouchama 2015). Indeed, studies modeling heat stroke have revealed increased AP‐1 signaling in skeletal muscle and increased IL‐6 transcript and protein expression in skeletal muscle and circulation (Welc et al. 2012, 2013). In contrast, heat stress is commonly caused by chronic hyperthermic exposure without a deliberate exercise intervention. Under these conditions 12 h of heat stress has been shown to cause suppressed IL‐6 protein abundance and rely heavily on NF‐*κ*B signaling (Ganesan et al. 2016).

It appears that increased free radical injury is a conserved response to heat stroke (Bouchama and Knochel 2002) and prolonged heat exposure (Mujahid et al. 2005, 2006; Azad et al. 2010; Montilla et al. 2014; Ganesan et al. 2017; Volodina et al. 2017) in skeletal muscle. Mitochondria both generate and are targets of free radicals, closely linking oxidative stress to mitochondrial dysfunction (Lee et al. 2012). Increased oxidative injury observed during heat stress raises the possibility of mitochondrial dysfunction and resultant mitochondrial inefficacy necessarily causing increased free radical (Kowaltowski and Vercesi 1999; Starkov 2008; Kikusato and Toyomizu 2013) and heat production (Poljsak 2011). That heat‐stress‐induced free radical damage and inflammation occur in oxidative muscle, but not glycolytic muscle (Montilla et al. 2014; Ganesan et al. 2016), further implicate mitochondria as central to heat‐stress‐mediated pathology.

Recent evidence has demonstrated autophagy is initiated in response to oxidative stress and mitochondrial dysfunction (Kowaltowski and Vercesi 1999; Chen and Gibson 2008; Xu et al. 2011), serving as a cytoprotective, prosurvival mechanism (Kiffin et al. 2006; Rabinowitz and White 2010). In addition, autophagy may remove large protein aggregates, which may be expected during heat stress (Burgio et al. 2000) in muscle where there is a sustained elevation in temperature without a corresponding change in heat shock proteins (Montilla et al. 2014; Selsby et al., unpublished observations). Indeed, increased autophagic signaling has been previously found in heat‐stressed germ cells (Zhang et al. 2012) and aged rat hepatocytes (Oberley et al. 2008) though may be dysregulated in heat‐stressed skeletal muscle (Ganesan et al. 2017). Hence, the purpose of this investigation was to determine the extent to which heat stress altered autophagic signaling in skeletal muscle from heat‐stressed pigs. Given the possibility of widespread cellular and mitochondrial dysfunction, we hypothesized that skeletal muscle cells would respond to heat stress by inducing autophagy.

## Materials and Methods

### Animal treatments

All procedures were approved by the Institutional Animal Care and Use Committee at Iowa State University. Data from these animals, including a detailed study design and animal treatments, have been previously published (Pearce et al. 2013a, 2013b, 2013c; Montilla et al. 2014). Briefly, to determine the extent to which heat stress caused skeletal muscle dysfunction female pigs (35 ± 4 kg; *n* = 5–6/group; Pig Improvement Company C22/C29 × 337) were kept at thermoneutral (TN) conditions (20 ± 1°C; 35–50% relative humidity) or subjected to continuous heat (35 ± 1°C; 20–35% relative humidity) for 1 day (HS1) or 3 days (HS3). After the treatment period pigs were sacrificed using the captive bolt technique and exsanguinated. The semitendinosus was collected and divided into red (STR) and white (STW) portions and frozen in liquid nitrogen for subsequent analyses.

### Immunochemistry

Approximately 50 mg of STR and STW muscle was powdered on dry ice using a steel mortar and pestle and then homogenized with a handheld homogenizer in 1.5 mL of protein extraction buffer (10 mmol/L sodium phosphate, pH 7.0, and 2% SDS). Following homogenization, samples were centrifuged at 1500*g* for 15 min at 4°C to remove cellular debris. Protein concentration was measured colorimetrically using the BCA assay (Pierce^®^ BCA microplate protein assay kit, Pierce, Rockford, IL). Samples were diluted to 4 mg/mL in loading buffer (62.5 mmol/L Tris, pH 6.8, 1.0% SDS, 0.01% bromophenol blue, 15.0% glycerol, and 5% *β*‐mercaptoethanol) and 10 *μ*L of each sample was loaded onto 420% gradient precast gels (Lonza PAGEr^™^ Gold Precast Gels). Protein was separated at room temperature for 30 min at 60 V followed by 50 min at 120 V and transferred to nitrocellulose membranes at 100 V for 60 min at 4°C. To assure equal loading, membranes were stained with Ponceau S and the resultant signal quantified. All quantification of staining was similar for all samples on all membranes. Ponceau S was removed and membranes were blocked in 5% dehydrated milk dissolved in Tris‐buffered saline containing 0.1% Tween 20 (TBST) for 1 h.

Membranes were incubated overnight at 4°C with primary antibody in 5% dehydrated milk TBST solution as follows (arranged as presented in results section): Adenosine Monophosphate Activated Protein Kinase (AMPK*α*) (Cell Signaling Technology, primary 1:1000, #2532, secondary 1:3000), Phospho‐AMPK*α* (Thr172) (CST, primary 1:1000, #2535, secondary 1:3000), Unc51‐like kinase 1 (ULK1) (CST, primary 1:1000, #8054, secondary 1:3000), Phospho‐ULK1(Ser555) (CST, primary 1:1000, #5869, secondary 1:3000), Phosphatidylinositide 3‐kinases (PI3K) Class III (CST, primary 1:1000, #3358, secondary 1:3000), Bcl‐2‐homology (BH)‐3 domain only protein (Beclin‐1) (CST, primary 1:1000, #3495, secondary 1:3000), p‐Beclin‐1 (Ser93) (CST, primary 1:500, #14717, secondary 1:500), microtubule associated protein light chain (LC3A/B) (CST, primary 1:1000, #12741, secondary 1:3000), Autophagy related (Atg) 5 (CST, primary 1:500, #12994, secondary 1:2500), Atg12 (CST, primary 1:750, #4180, secondary 1:3000), Atg16L1 (CST, primary 1:1000, #8089, secondary 1:2000), Atg7 (CST, primary 1:1000, #8558, secondary 1:3000), Atg3 (CST, primary 1:1000, #3415, secondary 1:3000), PTEN‐induced putative kinase 1 (PINK1) (CST, primary 1:500, #6946, secondary 1:2000), BCL2/Adenovirus E1B 19 kDa interacting protein 3‐like (Bnip3L/Nix) (CST, primary 1:500, #312396, secondary 1:2000), SQSTM1/p62 (Abcam, primary 1:500, ab109012, secondary 1:1000), Mitofusin‐2 (MFN2) (Abcam, primary 1:1000, ab50843, secondary 1:2000), Lamp2 (Abcam, primary 1:2000, ab101325, secondary 1:2000), Cathepsin‐L (Abcam, primary 1:1000, ab133641, secondary 1:2000), Cytochrome C (CYT C) (CST, primary 1:1000, #4280, secondary 1:3000), Cytochrome c oxidase IV (COX IV) (CST, primary 1:500, #4850, secondary 1:2000), Prohibitins 1 (PHB1), (CST, primary 1:1000, #2426, secondary 1:2000), Voltage‐dependent anion channel (VDAC), (CST, primary 1:1000, #4661, secondary 1:3000), Pyruvate dehydrogenase (PDH), (CST, primary 1:1000, #3205, secondary 1:3000), Succinate dehydrogenase (SDHA), (CST, primary 1:1000, #11998, secondary 1:3000), Heat shock protein 60 (HSP60), (CST, primary 1:1000, #12165, secondary 1:2000), total OXPHOS (CIII‐UGCRC2, CIV‐MTCO1, CII‐SDHB) (Abcam, primary 1:1000, ab110413, secondary 1:4000), Anti‐Sir2 (SIRT1) (Millipore, primary 1:1000, #07‐131, secondary 1:2000), Acetyl‐Histone H3 (Lys9) (H3K9) (CST, primary 1:1000, #9649, secondary 1:3000), PGC‐1 alpha + beta (PGC‐1*α*), (Abcam, primary 1:500, ab72230, secondary 1:1000), Estrogen‐related‐receptor (ERR*α*) (CST, primary 1:1000, #13826, secondary 1:3000).

Membranes were washed three times for 10 min in TBST and incubated in the appropriate secondary antibody in 5% dehydrated milk TBST solution for 1 h at room temperature (as described above). Membranes were washed three times for 10 min in TBST. Proteins were detected by enhanced chemiluminescence and X‐ray film, which were later scanned, and the blot signal was measured using Carestream software, Rochester, NY. The optical density of each band was determined and values for each sample were normalized to the mean of the thermoneutral samples for each blot. Secondary‐only controls were used to rule out nonspecific effects of secondary antibodies.

### qPCR

Powdered muscle was homogenized with TRIzol reagent and hand‐held homogenizers (Invitrogen; Carlsbad, CA; cat. no 15596), followed by centrifugation and extraction with chloroform. RNA was then precipitated from solution with ethanol. Total RNA was treated with DNase in order to remove any residual genomic DNA contamination (RNaseFree DNase set, Qiagen Inc., Valencia, CA; cat. no 79254) and purified with a spin column (RNeasy kit, Qiagen Inc.; cat. no 74106). RNA concentration was then determined with a Nanodrop (Thermo Scientific**,** Waltham, MA) and RNA was then reverse transcribed. Gene expression was measured through qRT‐PCR using SYBR green following the manufacturer's instructions (Qiagen Inc.; cat. no 204056). Transcript abundance of SQSTM1 (forward: 5′GTGTCCCCTTTCCTGTCTCA 3′; reverse: 5′CACACGTAGAACTCCACCCT 3′) was measured with the delta CT method and fold change calculated from the delta delta CT, with 18S rRNA (forward: 5′CTCTAGATAACCTCGGGCCG 3′ reverse: 5′ GTCGGGAGTGGGTAATTTGC 3′) as the control gene. Transcript abundance is represented as fold change during heat stress relative to thermoneutral. A no‐template control was used to verify nonspecific amplification was absent and all melting curves were inspected to assure single peak.

### Histology

For immunofluorescence analysis, 4% neutral‐buffered formalin‐fixed samples were sectioned at 10 μm thickness and mounted in the histology laboratory at the University of Iowa. Slides were deparaffinized using Citrisolv^™^ Hybrid Solvent (Fisher Scientific), rehydrated by 100%, 95%, and 80% ethanol for 5 min each, and followed by rinsing in distilled water. Antigen retrieval was performed by incubating slides in citrate buffer (Sodium Citrate: 29.4 gm/L ddH_2_O, Citric Acid: 21.1 gm/L ddH_2_O, pH 6, 1% Tween20) in laboratory microwave for 8 min and cooled for 20 min. Tissue sections were blocked in 5% BSA (Sigma Aldrich) in PBS for 90180 min. Primary antibody against Lamp2 (Abcam #109012; 1:100 dilution) and p62 (Abcam, #13524, 1:100 dilution) were applied to each section and incubated overnight at 4°C. Slides were washed thrice in PBS for 10 min each and incubated in fluorescent secondary antibody (Anti‐ rabbit IgG Fab2 Alexa Fluor 488, Cell signaling #4412S, 1:200 dilution and Goat anti‐ rat Rhodamine conjugate, Thermo Scientific, #31680, 1:100 dilution) for 1 hour at room temperature. Slides were washed three times in PBS for 10 min each and 4′,6‐diamidino‐2‐phenylindole (DAPI) stain was applied to each section. Importantly, negative controls including BSA/BSA, primary/BSA, and BSA/secondary‐treated slides did not have detectable signal. Multiple images/section were taken at 40x under identical imaging conditions using a Leica fluorescent microscope (*n* = 3/group).

### Statistics

Data were analyzed using SAS University Edition (SAS Institute Inc., Cary, NC). For analysis of treatment effects, data were analyzed using PROC MIXED, with treatments TN, HS1, and HS3 as fixed effects. Preplanned contrasts were used to evaluate the overall effects of heat compared to thermal neutral controls (TN vs. HS1 + HS3) using the CONTRAST statement of SAS. Significance was set at *P* < 0.05. Data are shown as means ± SEM.

## Results

### Phenotypic response

In order to cause heat‐stressed pigs were subjected to 1‐ or 3‐days of continuous environmental hyperthermia. A detailed report of the physiological response to this heat intervention has been published previously (Pearce et al. 2013a). In brief, rectal temperature was significantly increased from 39.3 ± 0.1°C in TN pigs to 40.8 ± 0.1°C in heat‐stressed animals. In addition, respiratory rate was increased from 54 ± 3 breaths per minute in thermoneutral animals to 107 ± 2 breaths per minute on average in heat‐stressed animals regardless of group (Pearce et al. 2013a).

### Markers of autophagosome membrane nucleation and elongation

**Figure 1:**
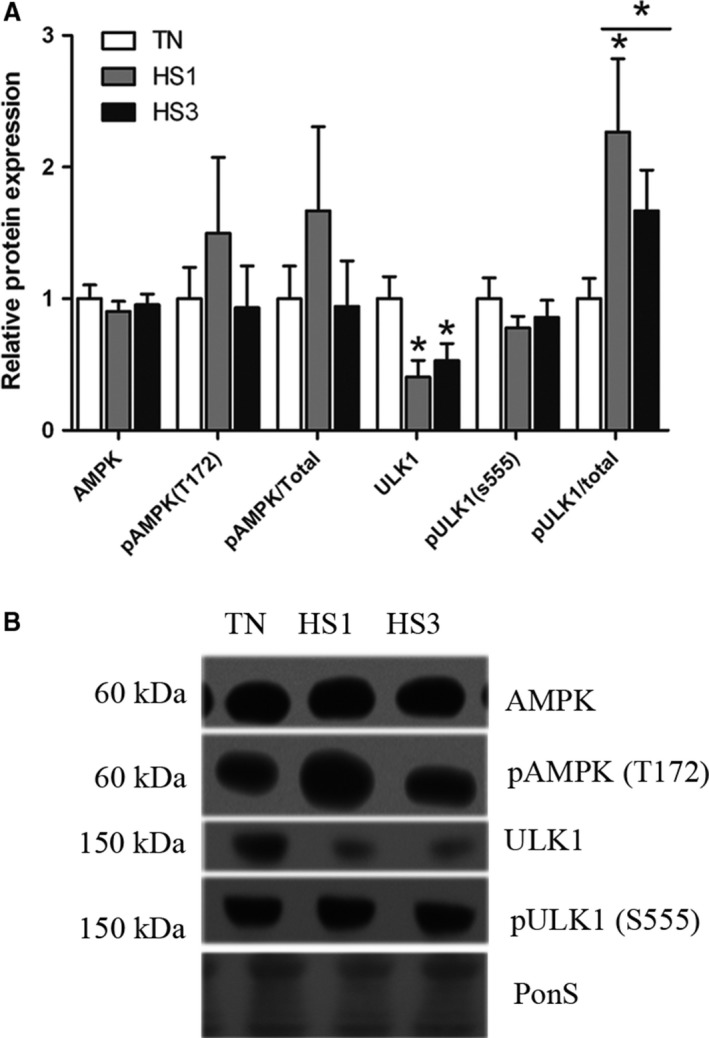
Activation of autophagy. (A) Following 1‐ and 3‐days of heat stress (HS; 35°C), markers of upstream activation of autophagy were assessed using Western blot. (B) Representative blots are included. Ponceau S stain (PonS) was used as a loading control. Values represent the mean ± SE for 4–6 gilts in each group. *indicates significantly different from TN (*P* < 0.05). TN, thermoneutral; HS, heat stress.

To determine the extent to which heat‐stress‐induced autophagy in skeletal muscle autophagic signaling was measured after 1‐ and 3‐days of heat stress in oxidative and glycolytic skeletal muscle. Autophagy can be initiated via AMPK*α* phosphorylation of ULK1. In STR we found relative protein abundance of total AMPK*α* and activated AMPK*α* (Thr172) (*P* = 0.5750) was similar between groups (Fig. 1A and B). Total ULK1 protein abundance was decreased 2.5‐ and 2.2‐fold in HS1 and HS3 compared to TN (*P* = 0.0197), however, abundance of pULK1 (Ser555), was similar between groups (*P* = 0.6510), though the ratio of pULK1 (Ser555)/total ULK1 was increased approximately twofold following HS compared to TN (*P* = 0.0239).

**Figure 2:**
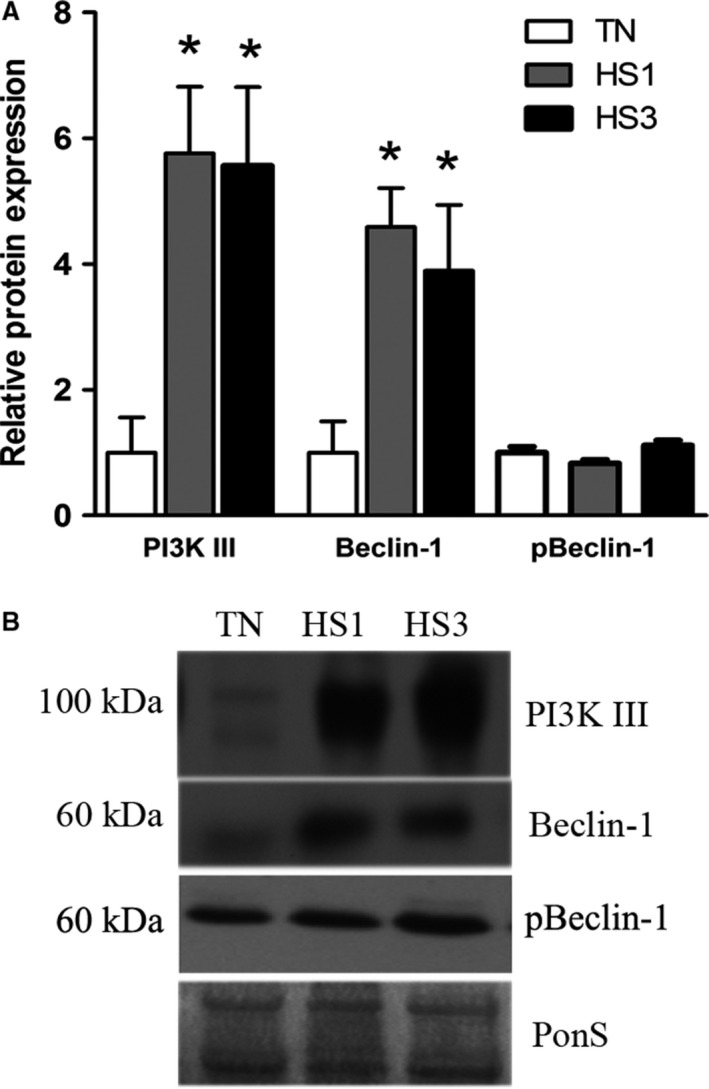
Autophagosome nucleation. (A) Following 1‐ and 3‐days of heat stress (HS; 35°C), markers of autophagosome nucleation were assessed by Western blot. (B) Representative blots are included. Ponceau S stain (PonS) was used as a loading control. Values represent the mean ± SE for 4–6 gilts in each group. *indicates significantly different from TN (*P* < 0.05). TN, thermoneutral; HS, heat stress.

Relative protein abundance of PI3 Kinase Class III and Beclin‐1 were measured as markers of membrane nucleation. Protein abundance of PI3Kinase Class III was increased 5.8‐ and 5.6‐fold (*P* = 0.0178) in HS1 and HS3 compared to TN (Fig. 2A and B). Beclin‐1 was increased 4.6‐ and 3.9‐fold (*P* = 0.0246) after 1‐ and 3‐days of heat stress compared to TN, however, phosphorylated Beclin‐1, indicative of activation by AMPK, was similar between groups.

**Figure 3:**
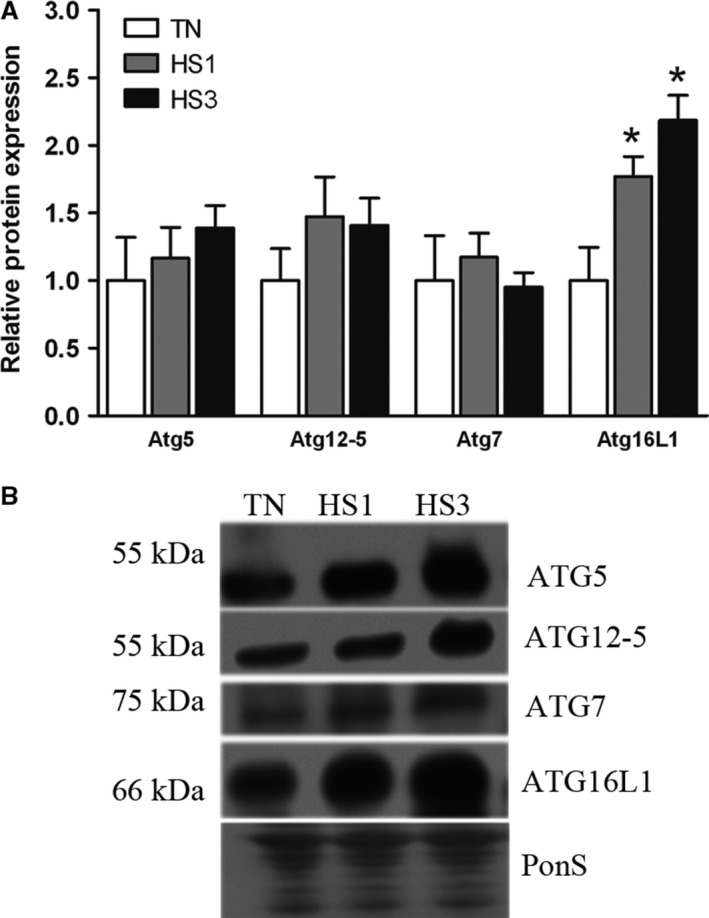
Autophagosome elongation. (A) Following 1‐ and 3‐days of heat stress (HS; 35°C), markers of autophagosome elongation were assessed by Western blot. (B) Representative blots are included. Ponceau S stain (PonS) was used as a loading control. Values represent the mean ± SE for 4–6 gilts in each group. *indicates significantly different from TN (*P* < 0.05). TN, thermoneutral; HS, heat stress.

**Figure 4:**
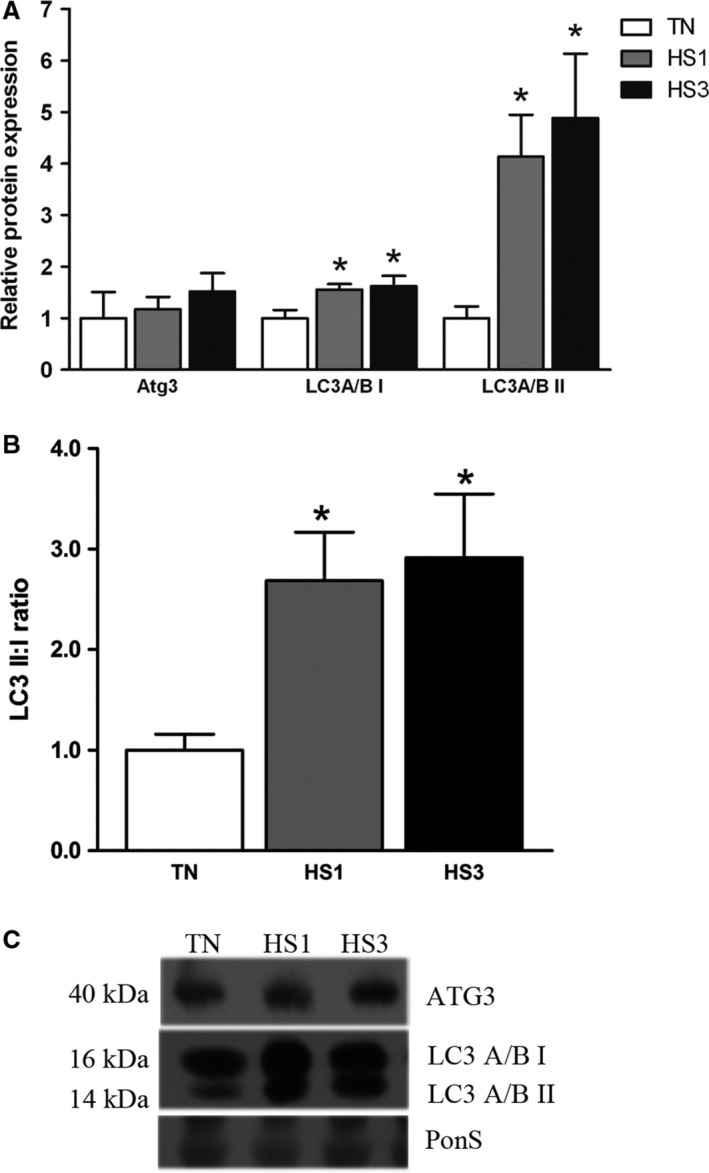
Sequestration. (A) Effects of thermoneutral conditions (TN; 21°C) or 1‐ and 3‐days of heat stress (HS; 35°C) on markers of autophagosome sequestration. (B) Calculated ratio of LC3A/B‐II to LC3A/B‐I. (C) Representative blots are included. Ponceau S stain (PonS) was used as a loading control. Values represent the mean ± SE for 4–6 gilts in each group. *indicates significantly different from TN (*P* < 0.05). TN, thermoneutral; HS, heat stress.

Elongation of the nascent autophagosomal membrane is controlled by two ubiquitin‐like conjugation systems, the Atg12/Atg5/Atg16L1 and LC3 system (Fujita et al. 2008; Wesselborg and Stork 2015; Zhang 2015). We found protein abundance of free Atg5 (*P* = 0.5354), the Atg12‐Atg5 complex (*P* = 0.4127), and Atg7 (*P* = 0.7216) were similar between groups, however, Atg16L1 was increased 1.8‐ and 2.2‐fold after 1‐ and 3‐days (*P* = 0.0040) of heat stress compared to TN (Fig. 3A and B). In the second ubiquitin‐like pathway involving LC3A/B, in addition to Atg 7, the conversion of LC3A/B to LC3A/B‐I and ultimately to LC3A/B‐II is dependent on Atg3 (Mizushima 2007; Fujita et al. 2008; Nakatogawa 2013; Wesselborg and Stork 2015). Relative abundance of Atg3 (*P* = 0.6111; Fig. 4A and C) was similar between groups. Relative protein abundance of LC3A/B‐I increased 1.6‐fold (*P* = 0.0443) after both 1‐ and 3‐days of heat stress (Fig. 4A and C). LC3A/B‐II was increased significantly by 4.1‐fold and 4.8‐fold (*P* = 0.0378) following 1‐ and 3‐days of heat stress (Fig. 4A and C). We also measured the relative conversion of LC3A/B‐I to ‐II, which was increased by 2.7‐ and 2.9‐fold in HS1 and HS3 compared to TN, respectively (*P* = 0.0498), suggesting an increased rate of autophagosome formation (Fig. 4B).

### Autophagic degradation

**Figure 5:**
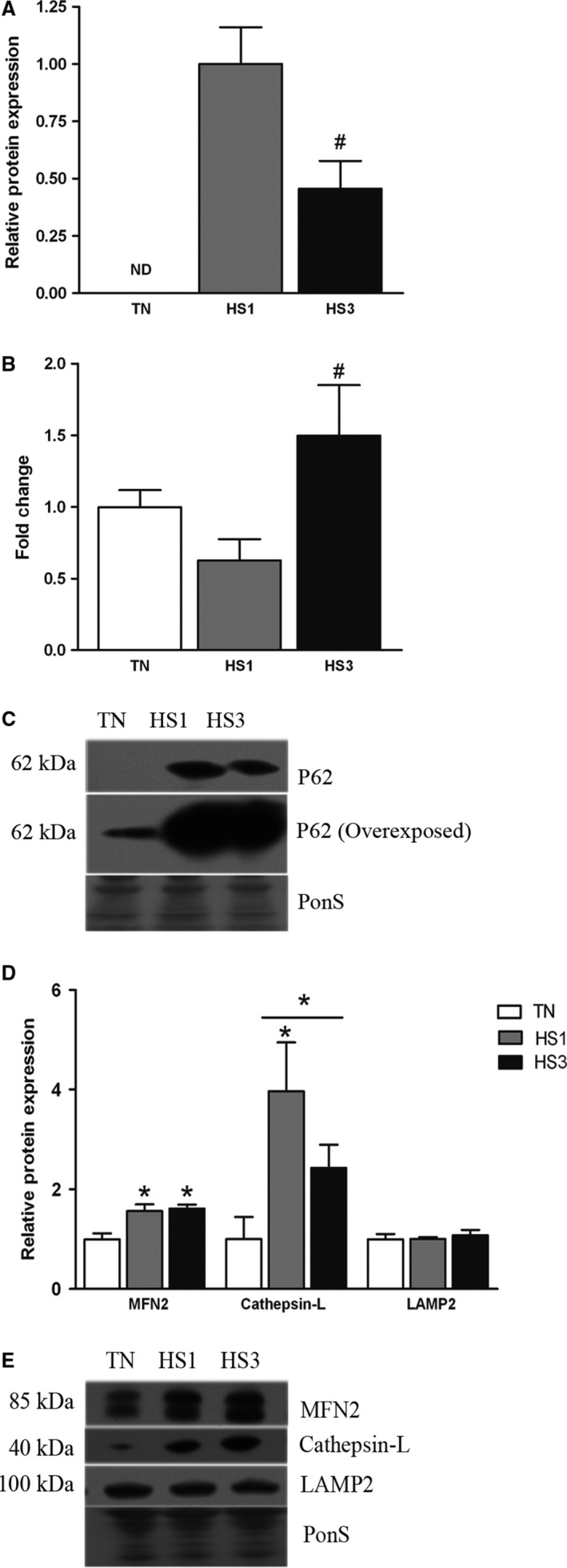
Markers of autophagic degradation. Effects of thermoneutral conditions (TN; 21°C) or 1‐ and 3‐days of heat stress (HS; 35°C) on (A) protein abundance of p62. Protein abundance was made relative to HS1. (B) Fold change of SQSTM1/p62 gene expression. 18s was used during analysis of gene expression as a control to identify fold changes. (C) Representative blots are included. Note, a blot was deliberately overexposed to demonstrate p62 was present in TN samples, however, this blot was not used in analyses. (D) Markers of lysosomal degradation. MFN2 is included because of its role in autophagosome/lysosome fusion. (E) Representative blots. Ponceau S stain (PonS) was used as a loading control. Values represent the mean ± SE for 4–6 gilts in each group. * indicates significantly different from TN (*P* < 0.05); #indicates significantly different from HS1 (*P* < 0.05). TN, thermoneutral; HS, heat stress.

To investigate autophagic degradation we measured relative abundance of p62, a selective autophagy receptor consumed during lysosomal degradation of autophagosomes (Lippai and Low 2014). P62 protein abundance was not detected in TN, though readily detectable in HS1 and HS3 indicating a dramatic increase in p62 accumulation and a reduction in autophagic degradation (Fig. 5A and C). Because p62 was not detectable in TN, data were normalized to HS1. P62 in HS3 was decreased by 2.2‐fold compared to HS1 (*P* < 0.05) (but was still readily detectable). Additional blots were overexposed to demonstrate p62 was present in muscle from TN pigs. We found transcript abundance of SQSTM1/p62 was similar between TN and HS1 and increased 2.4‐fold in HS3 compared to HS1 (Fig. 5B). These data indicate that heat stress caused a failure of autophagosome degradation.

Autophagosomes fuse with lysosomes to form autophagolysosomes, in part through the fusion protein Mitofusin2 (MFN 2), which was increased 1.4‐ and 1.6‐fold following 1‐ and 3‐days of heat stress (*P* = 0.0416), compared to TN (Fig. 5D and E). Additionally, we found a 3.9‐fold (*P* = 0.0061) and a numerical 2.4‐fold increase (*P* = 0.1400) in relative protein abundance of the lysosomal protease Cathepsin‐L after 1‐ and 3‐days of heat stress, respectively, compared to TN, and a 3.1‐fold increase in total heat stressed animals compared to TN (*P* = 0.0203; Fig. 5D and E). Relative abundance of the lysosomal membrane protein Lamp2 was similar between groups (*P* = 0.8025; Fig. 5D and E).

**Figure 6:**
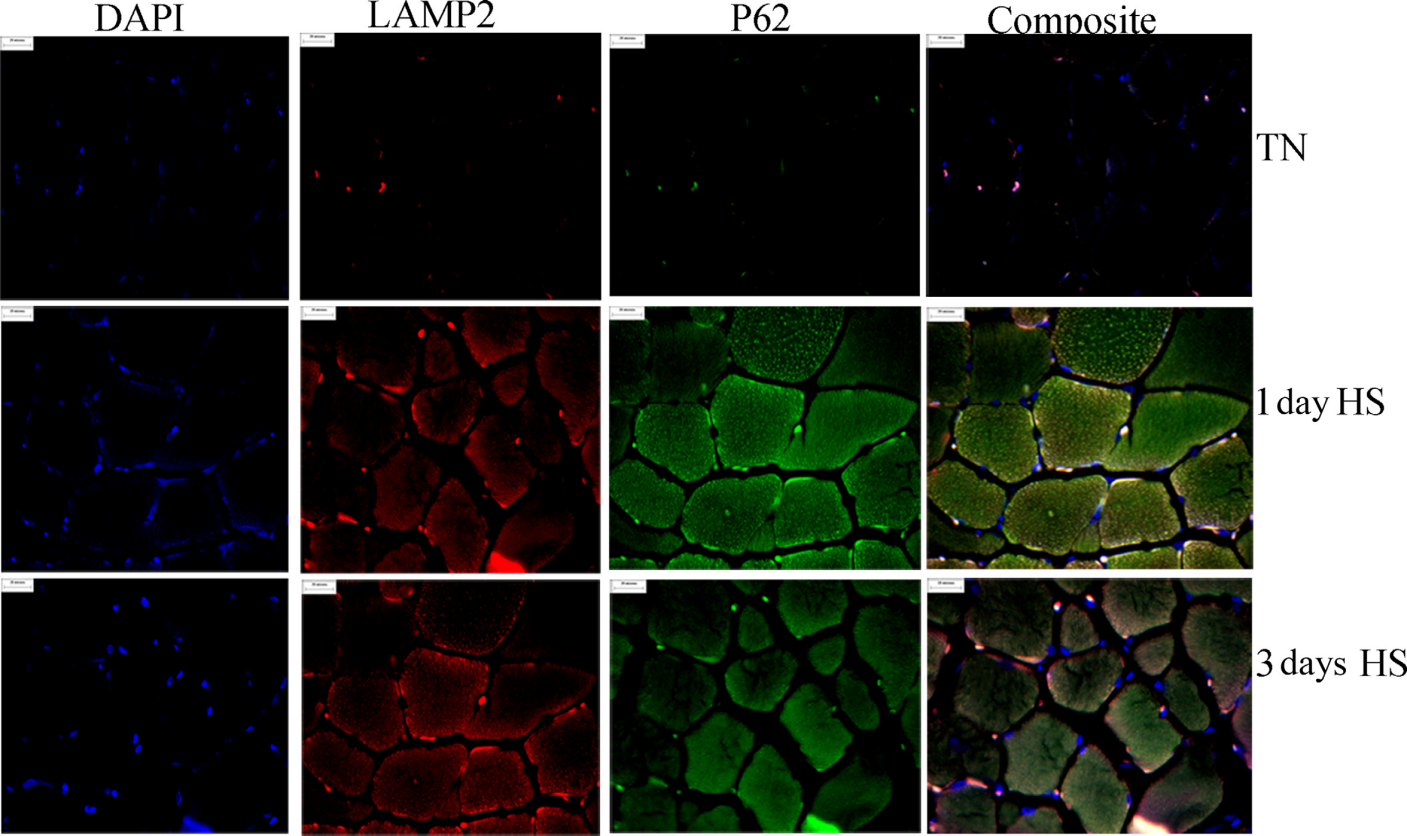
Autophagosome and lysosome localization. Effects of thermoneutral conditions (TN; 21°C) or 1‐ and 3‐days of heat stress (HS; 35°C) on localization of nuclei (blue), p62 (green), Lamp2 (red), and colocalization (yellow). P62 was used as a marker for autophagosomes and Lamp2 was used as a marker of lysosomes. All slides were treated and imaged under identical conditions.

To confirm accumulation of autophagosomes histological sections were probed with anti‐p62 antibodies (Fig. 6). Consistent with Western blot data, p62‐positive puncta were readily apparent in heat stressed samples. In addition, heat stress animals exhibited an increased abundance of the lysosomal marker Lamp2 within muscle cells. Satisfactory explanation for the incongruence between Western blot and immunohistochemical findings remains elusive though may reside in differing sensitivities of the measures and/or the localization of Lamp2 within the whole muscle. While there was a high degree of colocalization within muscle cells it was also apparent that within muscle cells there were p62‐positive puncta independent of Lamp2, particularly following 1 days of heat stress.

### Markers of mitophagy in heat stressed skeletal muscle

**Figure 7:**
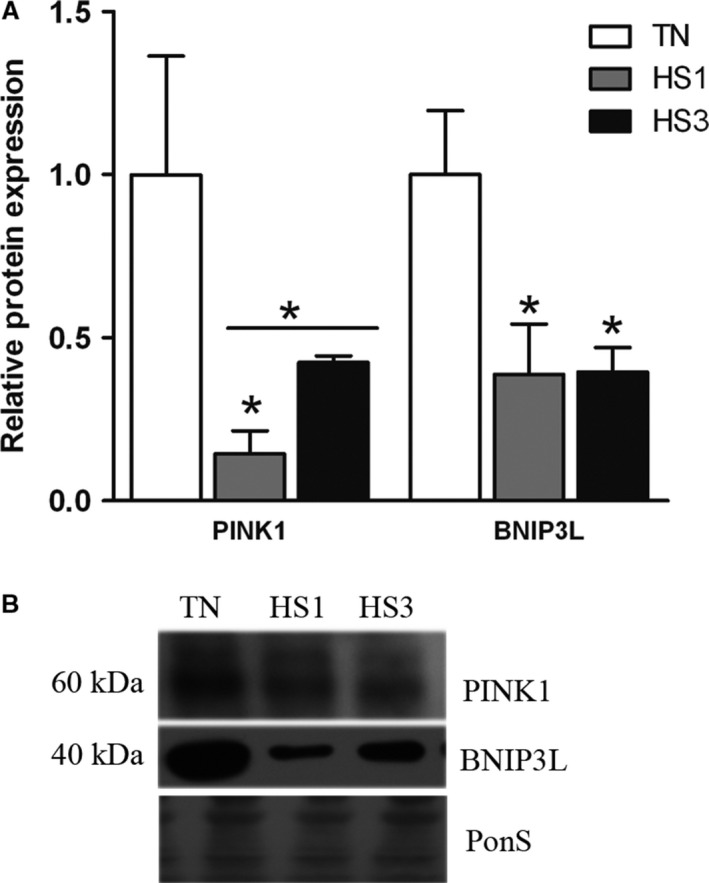
Markers of mitophagy. (A) Effects of thermoneutral conditions (TN; 21°C) or 1‐ and 3‐days of heat stress (HS; 35°C) on protein abundance of mitophagy markers using Western blot. (B) Representative blots are included. Ponceau S stain (PonS) was used as a loading control. Values represent the mean ± SE for 4–6 gilts in each group. * indicates significantly different from TN (*P* < 0.05). TN, thermoneutral; HS, heat stress.

To assess mitophagy we measured protein abundance of the mitophagy markers PINK1 and BNIP3L, which recruit and localize autophagic machinery to dysfunctional mitochondria (Wei et al. 2015). Relative abundance of PINK1 was decreased 7.0‐fold following 1‐day of heat stress (*P* = 0.0199) and numerically by 2.4‐fold following 3‐days of heat stress compared to TN (*P* = 0.0901; Fig. 7A and B). Comparison of thermoneutral to all heat stressed animals demonstrated a significant reduction in protein abundance of PINK1 (*P* = 0.0206). Additionally, BNIP3L was decreased 2.5‐fold (*P* = 0.0222) after both 1‐ and 3‐ days of heat stress (Fig. 7A and B).

### Heat stress leads to increased markers of mitochondrial content in oxidative muscle

**Figure 8:**
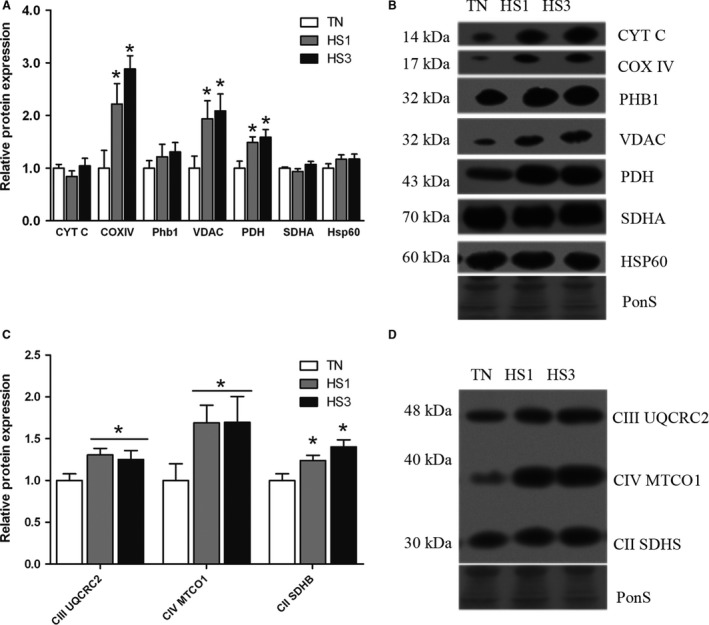
Markers of mitochondrial content. (A) Following 1‐ and 3‐days of heat stress (HS; 35°C) relative protein abundance of markers of mitochondrial content were assessed by Western blot. (B) Sample blots are included. (C) Markers of energy metabolism were also assessed. (D) Sample blots are included. Ponceau S stain (PonS) was used as a loading control. Values represent the mean ± SE for 4–6 gilts in each group. *indicates significantly different from TN (*P* < 0.05). TN, thermoneutral; HS, heat stress.

After finding markers suggesting diminished autophagic degradation and impaired mitophagy due to heat stress, we hypothesized that this would result in increased mitochondrial content. To assess this we measured abundance of proteins located in the mitochondrial membrane, matrix, and electron transport chain. We found that relative protein abundance of the inner mitochondrial membrane proteins Cytochrome C (*P* = 0.4417), SDHA (*P* = 0.1674) and PHB1 (*P* = 0.5537), and the outer matrix protein HSP60 (*P* = 0.2879) were similar between groups (Fig. 8A and B). However, we found a significant 2.2‐ and 2.8‐fold increase (*P* = 0.0069) in the inner mitochondrial membrane protein COXIV, a 1.9‐ and 2.1‐fold increase in the outer mitochondrial membrane protein VDAC (*P* = 0.0395), and a 1.5‐ and 1.6‐fold increase in the matrix protein pyruvate dehydrogenase (PDH) in HS1 and HS3, respectively, compared to TN (*P* = 0.0417; Fig. 8A and B). In addition, we measured components of the electron transport chain and found a 1.2‐ and 1.4‐fold increase (*P* = 0.0089) in Complex II Iron‐sulfur protein (IP) subunit of succinate dehydrogenase (SDHB) in HS1 and HS3 compared to TN and a 1.3‐fold increase (*P* = 0.0069) in relative abundance of ubiquinol‐cytochrome c reductase complex III (UQCRC2) when heat stressed animals were compared to thermoneutral (Fig. 8C and D). Cytochrome c oxidase subunit 1 (MTCO1) followed a similar pattern as SDHB and UQCRC2, with a 1.7‐fold increase in relative protein abundance of total heat stressed animals compared to thermoneutral, however, was similar between groups (*P* = 0.0883; Fig. 8C and D).

**Figure 9:**
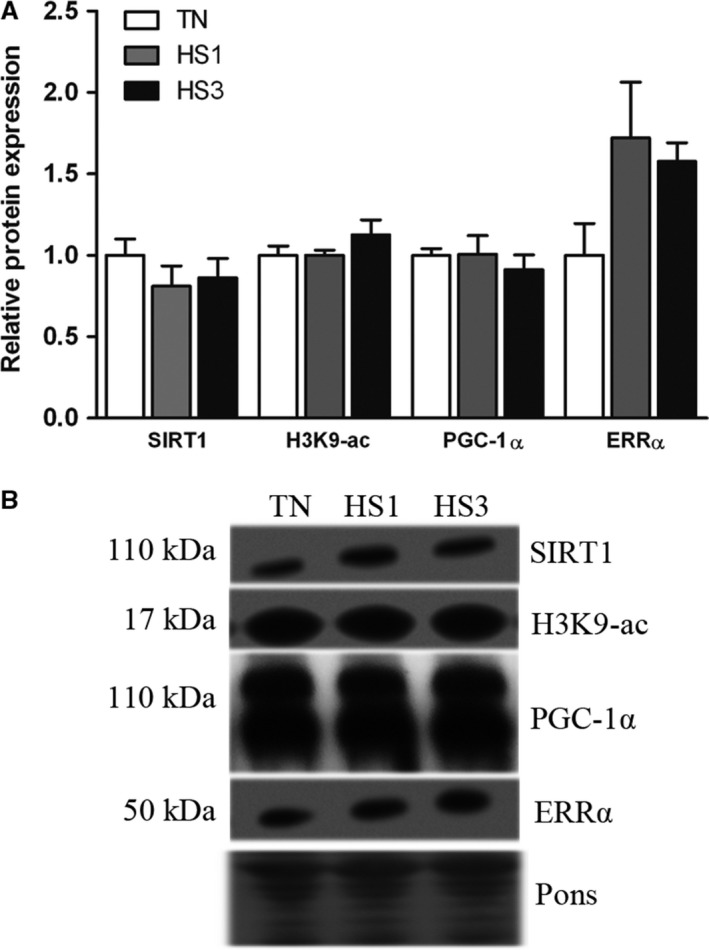
Markers of mitochondrial biogenesis. (A) Following 1‐ and 3‐days of heat stress (HS; 35°C) relative protein abundance of markers of mitochondrial biogenesis were measured by Western blot. (B) Representative blots are included. Ponceau S stain (PonS) was used as a loading control. Values represent the mean ± SE for 4–6 gilts in each group. * indicates significantly different from TN (*P* < 0.05). TN, thermoneutral; HS, heat stress.

Mitochondrial abundance could be maintained by increased biogenic signaling driven largely through PGC‐1*α*. Relative protein abundance of upstream PGC‐1*α* activator, SIRT1, was similar between groups (*P* = 0.4751) as was acetylation status of histone 3, reflective of SIRT1 activity (*P* = 0.3574; Fig. 9A and B). Relative protein abundance of PGC‐1*α* (*P* = 0.6850) was similar between groups though ERR*α* tended to be elevated with heat stress (*P* = 0.0977; Fig. 9A and B).

### Glycolytic muscle appears to be resistant to heat stress

**Figure 10:**
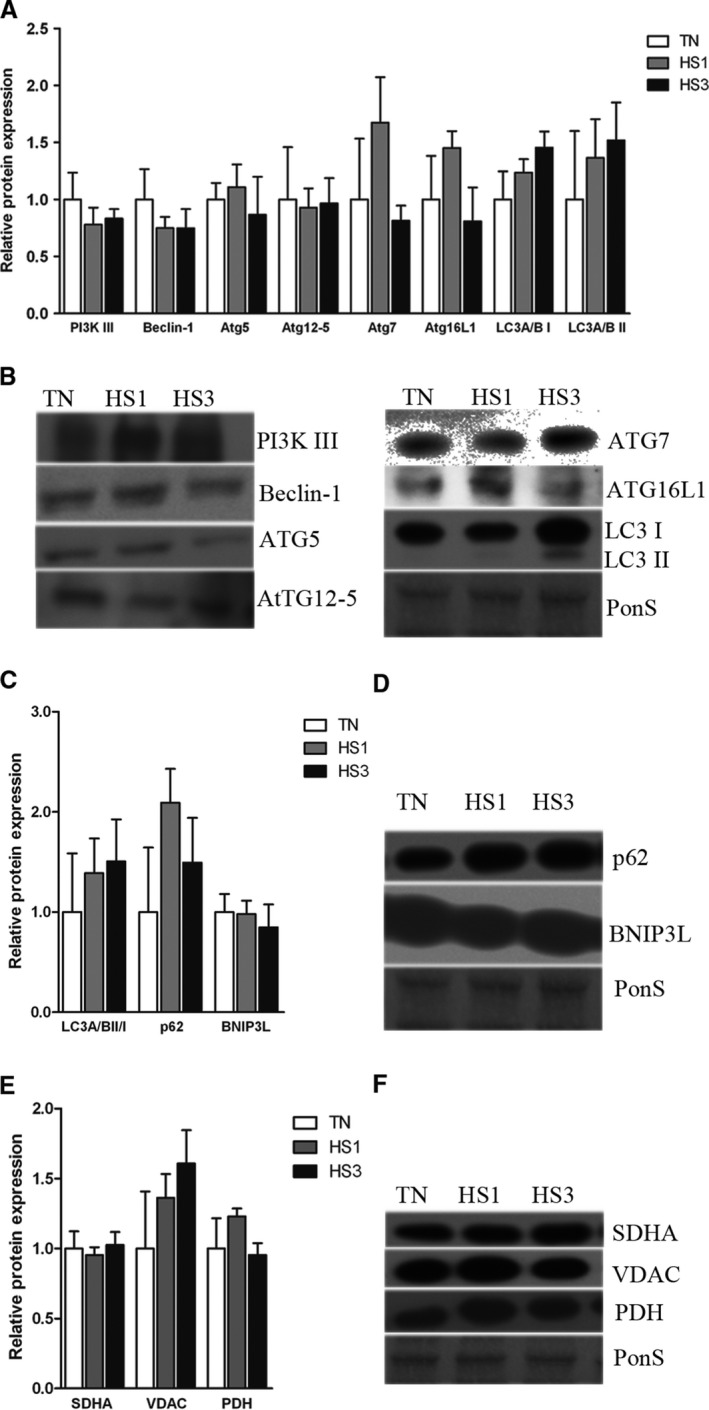
Effects of thermoneutral conditions and 1‐ and 3‐days of heat stress in STW glycolytic muscle. (A) Following 1‐ and 3‐days of heat stress (HS; 35°C) relative protein abundance was measured for autophagy signaling, (C) degradation, and (E) mitochondrial content by Western blot. (B, D, and F) Sample blots are included. Ponceau S stain (PonS) was used as a loading control. Values represent the mean ± SE for 46 gilts in each group. *indicates significantly different from TN (*P* < 0.05). TN, thermoneutral; HS, heat stress.

We also assessed autophagic signaling in glycolytic skeletal muscle. In contrast to oxidative muscle, in glycolytic muscle markers of autophagy (Fig. 10A and B), autophagic degradation (Fig. 10C and D), and mitochondrial content (Fig. 10E and F) were similar between groups.

## Discussion

Heat stress poses a threat to human and animal health. Gaining a mechanistic understanding of pathological changes caused by heat stress is a requirement for developing effective interventions. In oxidative skeletal muscle heat stress causes increased free radical injury (Mujahid et al. 2005, 2006, 2007; Montilla et al. 2014; Ganesan et al. 2017) and inflammation (Ganesan et al. 2016), but not in glycolytic muscle, suggestive of mitochondrial dysfunction and metabolic strain in heat‐stress‐mediated pathology. Given the potential linkage between heat stress and mitochondrial damage we hypothesized that in skeletal muscle heat stress would lead to increased autophagy initiation, autophagosome formation, and autophagosome degradation after 1‐ and 3‐days of constant hyperthermic exposure.

Counter to our hypothesis, upstream activation of autophagy was not stimulated by heat stress as AMPK and ULK1 phosphorylation was similar between groups. Despite robust elevations of PI3 Kinase class III and Beclin‐1 protein abundance by heat stress, phosphorylated Belcin‐1 was similar between groups. While relative abundance of Atg16L1, the limiting component of Atg12/Atg5/Atg16L1‐complex formation (Fujita et al. 2008), was increased during heat stress, Atg12/5 complex formation was similar between groups. Finally, LC3A/B‐I was significantly increased in heat stressed animals. Collectively, these data indicate that, despite a prooxidant intracellular environment, heat stress did not robustly stimulate autophagy in oxidative skeletal muscle. These data are in good agreement with recent findings in oxidative muscle following 12 h of heat stress (Ganesan et al. 2017).

Increased LC3A/B‐II abundance is often associated with increased autophagosome content. Likewise, increased LC3A/B‐II/I is often associated with increased flux. Importantly, in the context of this investigation, given that autophagy does not appear to be stimulated by heat stress, it is likely that accumulation of autophagosomes is caused by decreased degradation rather than a heat‐stress‐mediated increase in autophagosome formation, per se. This postulate is supported by increased protein abundance of p62, which is a selective autophagy receptor for damaged and tagged proteins and organelles (Bartlett et al. 2011; Lippai and Low 2014; Rogov et al. 2014). Importantly, p62 is consumed by lysosomes following fusion of autophagosomes with lysosomes. Hence, accumulation of p62 is reflective of suppressed autophagic degradation (Klionsky 2016).

In this investigation, the mechanism underlying this suppressed degradation cannot be empirically determined. Noteworthy, however, is the striking elevation in p62 positive puncta that do not colocalize with Lamp2 positive puncta particularly in HS1 raising the possibility of dysregulated fusion. Of interest, we recently found suppressed MFN2, an essential component of the fusion machinery, following 12 h of heat stress (Ganesan et al. 2017), along with additional markers of dysregulated autophagy. While more commonly associated with mitochondrial fusion, experiments with *mfn2* knockout mice have demonstrated its importance in autophagosome/lysosome fusion (Zhao et al. 2012). In this investigation, this limitation appears to be overcome as MFN2 protein abundance was increased in heat stressed groups. Free radical damage can disrupt the autophagy‐lysosome pathway and induce lysosomal permeabilization and destabilization (Zhang et al. 2014) serving to impede lysosomal degradation. Importantly, oxidative stress has been repeatedly found in heat‐stressed skeletal muscle (Mujahid et al. 2005, 2006, 2007; Ganesan et al. 2017; Volodina et al. 2017), including a previous publication using these samples (Montilla et al. 2014). Moreover, dysfunctional lysosomes may have a diminished capacity to fuse with autophagosomes (Mauvezin et al. 2015).

Impaired autophagosome degradation coupled with additional evidence including decreased markers of mitophagy and increased markers of mitochondrial abundance raise the possibility of blunted mitochondrial removal. Decreased abundance of PINK1, which recruits the E3 ubiquitin ligase Parkin to the mitochondria, provides a signal to selectively induce mitophagy (Dagda et al. 2009; Gegg et al. 2010; Kawajiri et al. 2010). Additionally, BNIP3L has been shown to induce mitophagy and resultant removal of dysfunctional mitochondria (Quinsay et al. 2010; Lee et al. 2011; Rikka et al. 2011). The reduction in these two proteins coupled with increased markers of mitochondrial abundance independent of biogenic signaling suggests preservation of damaged mitochondria, which could contribute to a prooxidant intracellular environment. In addition, it would cause decreased mitochondrial function as has been previously found following heat stress (Baumgard and Rhoads 2012).

Our results in glycolytic muscle are in good agreement with our previous work demonstrating a greater resistance to heat‐stress‐induced free radical injury (Montilla et al. 2014) and inflammatory signaling (Montilla et al. 2014; Ganesan et al. 2016) than oxidative muscle. Given the profound changes in oxidative muscle it is likely that mitochondria are critical to heat‐stress‐mediated muscle dysfunction. In glycolytic muscle the lower mitochondrial content may provide resistance to heat‐stress‐mediated pathologies.

## Limitations

In this investigation, we studied heat stress in a large animal model. As our intent was to address a significant human and agricultural problem, a large animal model was required. Given this, it was not feasible to include groups treated with inhibitors that are commonly used in studies of autophagic flux in cell culture and rodent models. Because of this we were not able to assess flux directly hence, while our data point strongly to autophagosome accumulation due to decreased degradation, we cannot eliminate the possibility that some portion of autophagosome accumulation is due to increased flux. In future experiments we expect to utilize cell culture and rodent models to facilitate measurement of autophagic flux and will plan to include measures of mitochondrial function.

## Perspectives and Significance

In total these data indicate heat‐stress‐mediated suppression of autophagic degradation potentiating an accumulation of autophagosomes in oxidative skeletal muscle. The combination of suppressed autophagic degradation and mitophagy, and increased markers of mitochondrial content raises the possibility of aberrant preservation of dysfunctional mitochondria, which would further contribute to a pathologic intracellular environment.

## Conflict of Interest

The authors have no conflicts to report.
